# The role of response conflict in concealed information detection with reaction times

**DOI:** 10.1038/s41598-023-43779-3

**Published:** 2023-10-19

**Authors:** Nathalie klein Selle, Barak Or, Ine Van der Cruyssen, Bruno Verschuere, Gershon Ben-Shakhar

**Affiliations:** 1 Department of Criminology, Bar-Ilan University, Ramat Gan, Israel; 2 Department of Psychology, Hebrew University of Jerusalem, Jerusalem, Israel; 3 Department of Clinical Psychology, University of Amsterdam, Amsterdam, The Netherlands

**Keywords:** Psychology, Human behaviour

## Abstract

The concealed information test (CIT) presents various probe (familiar) items amidst irrelevant (unfamiliar) items. When the probe items appear, reaction time (RT) slows down. This RT-CIT effect has been accounted for by a conflict resulting from the need to deny familiarity of the familiar probes. The present pre-registered study (*n* = 292) examined whether response conflict is sufficient to account for the RT-CIT effect, using city and name items. Specifically, we compared the common *conflict* condition, where the response buttons emphasized familiarity of CIT items (“unfamiliar” versus “familiar”), to a novel *no conflict* condition, where the buttons emphasized categorical membership (“city” versus “name”). In line with our expectations, the RT-CIT effect was substantially stronger in the conflict condition; yet, it remained significant even in the no conflict condition. This implies a critical role for response conflict, but also suggests that other mechanisms (e.g. orientation to significant stimuli) may contribute to the RT-CIT effect.

## Introduction

We all keep secrets. Some are trivial and harmless, while others are severe and may even threaten society. Imagine, for example, a kidnapper hiding the location of his victim, or a wanted terrorist concealing his true identity. Fortunately, researchers have developed a scientific method to uncover hidden knowledge, the so-called Concealed Information Test (CIT)^1,2^. This test involves the presentation of various significant details (e.g. your last name), labeled probe items, as well as irrelevant items (e.g. other last names). Initial CIT studies have relied on various autonomic nervous system measures, such as skin conductance^1,3–5^, while subsequent studies started measuring reaction time (RT)^6–10^. To validly measure RT, a third stimulus type, called targets, was added. These target items help to ensure stimulus processing by requiring a differential response. Specifically, examinees are typically instructed to press ‘yes’/‘familiar’/‘recognized’/‘target’ for the targets only versus ‘no’/‘unfamiliar’/‘non-recognized’/‘non-target’ for all other stimuli (probes and irrelevants). Many studies using this method have demonstrated longer RTs for probes than irrelevants, i.e. shown the RT-CIT effect.

The RT-CIT effect is a highly valid and stable phenomenon. A recent meta-analysis by Suchotzki et al.^11^ combined the results of 23 RT-CIT studies and found a large standardized difference between probes and irrelevants (i.e. Cohen’s *d* = 1.30; 95% CI [1.06; 1.53]). Although this effect size is impressive, scientific validity also requires a theoretical understanding of the CIT effect. A structural analysis of the RT-CIT has highlighted the importance of stimulus–response incompatibility^12^. Notably, only probe items exhibit such incompatibility, as the required “unfamiliar” response is incompatible with the automatically induced “familiar” response. This incompatibility is assumed to create a conflict. To resolve this conflict, participants must inhibit their tendency to respond “familiar” to the probes, which requires time and consequently may account for the longer RTs to probes versus irrelevant items (i.e. the RT-CIT effect). Indeed, the main theory of the RT-CIT effect suggests that it reflects an underlying *response conflict*^12–14^*.* Various studies have found empirical support for the conflict account of the RT-CIT effect. Suchotzki et al.^15^, for example, manipulated overt deception by instructing participants to admit knowledge of half of the probe items and deny knowledge of the other half. While overt deception was not necessary for the skin conductance response (SCR)-based CIT effect, it was crucial for the RT-CIT effect, suggesting that this measure reflects response inhibition (that is, the resolution of response conflict). Interestingly, within the same study, the authors also measured brain activity and found that brain regions associated with inhibition, including the right inferior frontal gyrus, were only activated when participants answered “NO” (i.e. denied knowledge of the probe items), again supporting the role of response conflict. Suchotzki et al.^16^ took another approach to test the role of response conflict. Specifically, as conflict results from the fact that one needs to deny familiarity with the known probe items, conflict should be stronger when one relies more heavily on familiarity. Hence, the authors aimed to increase familiarity-based responding by: (1) Increasing the number of different target items. As familiarity is a shared feature of all targets, a stronger reliance on familiarity should make it easier to identify the different targets; (2) Using target items that were already familiar to the participant before the test. Again, a stronger reliance on familiarity, should make it easier to identify the targets. Both of these manipulations increased the RT-CIT effect, suggesting that response conflict plays a critical role in the RT-CIT. Most recently, using analog gaming keyboards that are sensitive to very subtle finger movements, Koller et al.^17^ found that probes elicit more partial errors than irrelevant items. The fact that probes elicit an initial tendency to press the incorrect response key fits well with the conflict account (see also Ref.^14,18,19^).

Taken together, it has been suggested that the enhanced RTs to probe items reflect response conflict. However, few findings do not support this reasoning^20,21^; for example, some studies found no relation between executive functioning and the RT-CIT effect^15,17,22^. Moreover, it is unclear whether conflict theory is a sufficient account of the RT-CIT effect. For example, orientation to the significant items, which accounts for the SCR-CIT effect^23–25^, may also contribute to the enhanced RTs to probe items. It should be noted that the size of the orienting response is modulated by stimulus significance as well as stimulus novelty^26,27^. Importantly, for knowledgeable individuals, probe items are both significant and novel (i.e. presented in a relative smaller frequency). Hence, probes should elicit an enhanced orienting response which may induce a brief period of motor inhibition (as manifested by longer RTs), in favor of a more rigorous analysis of the probes (see Ref.^28,29^). Indeed, few studies have shown that an orienting stimulus can slow performance using different tasks^30,31^. Moreover, few CIT studies have directly shown that the RT-CIT effect is modulated by stimulus salience – i.e. more significant (salient) probes such as one’s own name elicited a stronger RT-CIT effect than less significant (salient) probes such as one´s favorite color or animal^32,33^. This carefully suggests that, in addition to conflict, also orienting may play a role in the RT-CIT effect.

The present study compared two conditions that used the exact same stimuli, but differed in their task instructions which should affect the strength of experienced response conflict (for a similar approach see Ref.^34^). In the “conflict” condition, participants were instructed to judge the CIT stimuli on familiarity, by pressing buttons with the captions “familiar” versus “unfamiliar”, as is common in RT-CIT research. Responding “unfamiliar” to the familiar personal details is presumed to create response conflict. In the “no conflict” condition, participants were instructed to judge the stimuli on semantic category membership, by pressing buttons with the captions “city” versus “name”. Classifying a probe according to its semantic category (e.g. classifying one’s city of birth as being a city) should not create response conflict. Thus, while the *conflict* condition induced conflict, the *no conflict* condition did not involve any conflict. Altogether, the obtained results may expand current CIT theory and offer new ways in which the RT-CIT may be improved.

## Method

This study was approved by the Ethics Review Board of the Criminology department of Bar-Ilan University (BIU; March 24th, 2022) and was performed in accordance with the relevant guidelines and regulations. The experimental design and procedure were pre-registered on: https://aspredicted.org/e7ys3.pdf.

### Deviations from preregistration

As indicated in the pre-registration (see https://aspredicted.org/e7ys3.pdf), we adopted a Bayesian procedure to determine the sample size. Specifically, we collected a first batch of data which included at least 100 participants. We would stop data collection, once substantial evidence was obtained for either the alternative hypothesis (the 2 conditions differ), or the null hypothesis (the 2 conditions do not differ). Substantial evidence is defined by the Bayes factor (BF), namely, if either BF > 5 or BF < 0.2. Otherwise, we would add batches of *N* = 100 until we reached substantial evidence for either hypothesis or when *N* = 300 participants (with *N* being the number of participants tested, before exclusions are made). After the second batch we obtained substantial evidence for the alternative hypothesis (i.e. the conflict and no conflict conditions differed). However, due to a computational error of the BF, we collected another batch of data. Furthermore, it should be noted that although we preregistered to base our conclusions on BFs, we also report *p* values and Cohen’s *d* values, as requested by one of the reviewers.

We aimed to obtain the data for batch 1 by using BIU students (as part of their “Lie detection” course). However, as we only reached *N* = 30, we obtained the rest of this batch using the Prolific online research platform. Moreover, after obtaining the data of batch 1, we noticed that participants rated the irrelevant items as more significant than the probe items. Hence, for the subsequent two batches, we added the rating scale labels to the actual scales (1 = *not significant at all*, 9 = *extremely significant*), in addition to verbally describing them in the instructions. Finally, for batches 2 and 3 we also clarified in the instructions that favorite city cannot equal city of birth (these items served as probes in the CIT; see “Procedure”).

### Participants

The initial batch of data (*N* = 147, 59% female) was obtained by using Bar-Ilan University students and the Prolific online research platform (www.prolific.com). On Prolific, native Hebrew speaking participants were granted 1.5 British Pound (£) for participation. Participants’ average age was 28.67 years (*SD* = 7.7, range = 19–55). From this initial sample, 13 participants were not approved by prolific (e.g. the session timed out), 21 participants did not complete the CIT and 8 participants made at least 50% errors (in the CIT) to at least one of the 3 stimulus types (i.e. probes, irrelevant, targets). After excluding these participants, we remain with *N* = 105 (147–13–21–8).

The second batch of data (*N* = 160, 46% female) was obtained using the panel4all online research platform (www.panel4all.com) where native Hebrew speaking participants were granted a voucher of 10 NIS. Participants’ average age was 40.29 years (*SD* = 13.4, range = 18–72). From this initial sample, 57 participants did not complete the CIT, 11 participants performed the CIT more than once (as indicated by their unique panel4all ID number) and an additional 3 participants made at least 50% errors (in the CIT) to at least one of the 3 stimulus types. After excluding these participants, we remain with *N* = 89 (160–57–11–3).

The third batch of data (*N* = 172, 43% female) was also obtained using the panel4all online research platform (and native Hebrew speaking participants were again granted a 10 NIS voucher). Participants’ average age was 39.24 years (*SD* = 15.4, range = 18–73). From this initial sample, 57 participants did not complete the CIT, 10 participants performed the CIT more than once and an additional 7 participants made at least 50% errors (in the CIT) to at least one of the 3 stimulus types. When excluding these participants, we remain with *N* = 98 (172–57–10–7).

In sum, the final sample included in the main analyses involved 292 participants. There were 131 in the *conflict* condition (average age = 32.05 years; 53% female), and 161 in the *no conflict* condition (average age = 33.23; 53% female). We report on the full sample in the main analyses, but also report the results without the last batch (i.e. the preregistered sample size; that supports the main analyses). The Bayesian procedure as well as exclusion criteria were pre-registered.

### Design

This experiment includes one between-subjects factor: response conflict (“conflict” versus “no conflict”). In addition, to avoid stimulus-specific effects, we counterbalanced the type of items used (probes being either cities or names). Hence, participants were randomly allocated to 1 of 4 conditions: (1) conflict with cities as probes; (2) conflict with names as probes; (3) no conflict with cities as probes; (4) no conflict with names as probes. In the statistical analyses, we compared the “conflict” conditions (1, 2) with the “no conflict” conditions (3, 4).

### Procedure

The experimental task (online RT-CIT) was programmed with Inquisit 6 (see script on https://osf.io/8p2b4/) and took approximately 15 min. Participants were required to download Inquisit and run the RT-CIT test by using a unique link that was either sent to them directly or placed on one of the research platforms (i.e. Panel4all or Prolific). Only participants who were at least 18 years old, and whose native language was Hebrew, could participate. After reading and approving an informed consent form, participants were requested to complete various demographic details: i.e. gender, age, native language, and education.

#### Stage 1: item selection

Participants were asked to fill in two personal details: Either their last name and mother's name or their city of birth and favorite city, which were used as probes in the CIT. Thus, participants’ probe items were either 2 names or 2 cities. With regards to favorite city, Israeli born participants were instructed to choose a city outside of Israel. Participants born abroad were requested to pick a favorite Israeli city. Then, participants were presented with two item-lists; each list contained either 16 names (if the probes were names) or 16 cities (when the probes were cities). Participants were asked to mark all items that hold any special significance to them (e.g. names of close relatives and friends) as well as items that were similar to the probes (e.g. Nitza and Nitzana). Participants were not able to select more than 12 (from 16) items. Importantly, the irrelevant items (for the CIT) were chosen randomly from the unmarked items.

#### Stage 2: CIT

After finishing stage 1, participants were introduced to the RT-CIT. Specifically, they were explained that they would undergo a “recognition test” in which they have to try and conceal their personal details (i.e. either the 2 names or the 2 cities). To increase motivation, participants read a short paragraph which stated that the upcoming task is difficult, and that only highly intelligent people with a strong willpower can successfully conceal (see Ref.^35^). In addition, to become familiar with the target items, participants read a short paragraph highlighting these items (e.g. Who has not heard about the city of Milan, which is located in northern Italy and known for its great wealth? And of course, there is no one who does not know the city of Caesarea that was established 2000 years ago by the Roman Empire!). Note that if the probe and irrelevant items were names, targets were cities (i.e. “Caesarea” and “Milan”), and vice versa, when probes and irrelevants were cities, the targets were names (i.e. “Golda” and “Nahari”).

Importantly, in the *conflict* condition, participants were instructed to judge the CIT stimuli on familiarity, and to press buttons with the captions “familiar” (for targets) versus “unfamiliar” (for probes and irrelevants). As explained previously, the requirement to respond “unfamiliar” to personal details is presumed to create response conflict. In the *no conflict* condition, participants were requested to judge the stimuli on semantic category membership and the buttons had the captions “city” versus “name”. Classifying a probe according to its semantic category should not create response conflict. Nevertheless, the same two keyboard buttons were used in both conditions: “E” was captioned with “familiar” or “name”, “I” was captioned with “unfamiliar” or “city”.

The RT-CIT was operated according to the multiple probes protocol (MPP). This means that both categories (either the 2 names or the 2 cities) were intermixed in each block of the CIT (there were 4 blocks in total). Each category included one probe, one target and 4 irrelevant items. Thus, both categories included 2 probes, 2 targets and 8 irrelevant items (12 items in total). Each item was presented 6 times in each block. Thus, each block contained 72 items (12 × 6 = 72), and the whole experiment contained 288 items (72 × 4 = 288). The order of items’ presentation was determined randomly, with the following restriction: two consecutive presentations of the same item were not allowed (e.g. if the first item is London, the following item cannot be London as well). Each item appeared verbally on a white background in the middle of the screen. The item disappeared when participants pressed either the “E” or “I” button or when the 1500 ms presentation time elapsed. The next stimulus appeared on the screen after a short and randomly varying delay; the inter-stimulus-interval (ISI) between two items was either 250 ms, 500 ms, or 750 ms. During the interval between two items a symbol of a plus was presented in the middle of the screen. If participants pressed the wrong button, they saw the word “WRONG” in red above the item for 200 ms. Moreover, if 800 ms passed since the item appeared and no button was pressed, the words “TOO SLOW” appeared in red above the item.

Before the actual RT-CIT, participants underwent three successive practice phases to familiarize them with the CIT procedure. Although the practice-phase items differed from those in the real test, they were the same across practice phases and participants (either cities or names, depending on the experimental condition). Item-order was also the same for each participant, but differed between practice phases. In the first practice phase, items remained on the screen until one of the two available buttons (“E” or “I”) was pressed. When pressing the wrong button, participants received “WRONG” feedback. In the second practice phase, items remained on the screen until a button was pressed or until 1500 ms had passed. Just as in the first practice phase, the "WRONG" feedback was applied, but not the “TOO SLOW” feedback. In the third practice phase, which was identical to the test phase, participants also received "TOO SLOW" feedback when they did not press any button within 800 ms. In order to pass the practice phases, participants had to meet various criteria: (1) for phases 1–3, no more than 50% errors (i.e. wrong button pressings) should be made, (2) for phases 2–3, the mean RT should also be higher than 150ms, and (3) for phase 3, the mean RT should also be lower than 800ms. If participants did not meet these criteria, they received feedback about their performance (e.g. “Sorry, you failed this practice phase. Please repeat the training”) and had to perform the practice phase again (each phase could be executed no more than 2 times).

#### Stage 3: ratings

After the CIT, participants were asked to rate the significance level of the 2 probes and 8 irrelevant items (4 from each category), on a scale from 1 (= *not significant at all*) to 9 (= *extremely significant*). Then, participants were asked to rate their motivation to conceal during the CIT, on a scale from 1 (= *not motivated at all*) to 9 (= *extremely motivated*). Finally, participants were asked to write a few sentences about whether or not they tried to use countermeasures (i.e. ways to conceal their identity).

## Results

All data were pre-processed using Matlab R2023a (The MathWorks, Natick, MA) and analyzed using JASP software (version 0.11.1). Figure 2 was created using R software (version 4.0.5). The data and analysis scripts can be accessed at: https://osf.io/8p2b4/.

### Non-preregistered analyses: subjective ratings

Before analyzing the key outcome variable (RT-CIT effect), we analyzed the ratings which were obtained after the CIT. First, we analyzed participants’ motivation to conceal their identity during the CIT. The motivation to conceal was high and did not differ between the no conflict (*M* = 4.52, *SD* = 2.57) and conflict (*M* = 4.30, *SD* = 2.55) conditions, BF_01_ = 5.80.

Second, we analyzed the self-reported significance of probe and irrelevant items. Specifically, we computed a “significance difference score” (i.e. significance of probes minus significance of irrelevants) and compared this score across conditions. This analysis supported the null hypothesis of no difference; BF_01_ = 3.09. However, the “significance difference score” was in the opposite direction than expected. Namely, the significance of probes (*M* = 4.20, *SD* = 3.12) was lower than that of irrelevants (*M* = 5.28, *SD* = 2.75), BF_10_ = 17.62. As the probes were highly personal items (e.g. last name), which have been validated in many earlier experiments^24,36^, it suggests that our participants did not fully understand the instructions of this part of the experiment. This was already apparent in the first batch. Hence, for the subsequent two batches, we added the rating scale labels to the actual scales (1 = *not significant at all*, 9 = *extremely significant*), in addition to verbally describing them in the instructions. Unfortunately, this did not change participants’ rating behavior (see “Discussion”).

### Preregistered analysis: main hypothesis

For the main analysis, we computed for each participant the RT-CIT effect: i.e. the mean RT of probes minus the mean RT of irrelevants. These averages were calculated after excluding: (1) each error of pressing the wrong button; (2) each button press under 150ms; (3) each button press above 800ms (see https://aspredicted.org/e7ys3.pdf).

Then, a Bayesian independent samples *t*-test compared the RT-CIT effect in the “conflict” (*M* = 35.84, *SD* = 34.14) and the “no conflict” (*M* = 23.40, *SD* = 32.04) conditions, see Table 1. This test revealed a substantial difference: BF_10_ = 16.27 (Mdifference = 12.44 [4.84, 20.04], p = 0.002, Cohen’s d = 0.38), implying that the alternative hypothesis (i.e. RT-CIT effect in the conflict condition ≠ RT-CIT effect in the no conflict condition) is about 16 times more likely than the null hypothesis (i.e. there is no between-condition difference).

**Table 1:** Mean RTs in conflict and no conflict conditions, along with the RT-CIT effect, separate for the two types of probes (names and cities).

Probe type	Conflict				No conflict			
	M (SD) probes	M (SD) irrelevants	RT-CIT effect (M_probes_–M_irrelevants_) with 95% CI	Cohen’s *d* with 95% CI	M (SD) probes	M (SD) irrelevants	RT-CIT effect (M_probes_–M_irrelevants_) with 95% CI	Cohen’s *d* with 95% CI
Names + cities	508.72 (51.30)	472.88 (43.96)	35.84 [29.99; 41.68]	1.05 [0.84; 1.26]	511.24 (59.90)	487.85 (55.28)	23.40 [18.45; 28.35]	0.73 [0.56; 0.90]
Names	522.84 (52.45)	480.19 (45.14)	42.65 [33.98; 51.31]	1.24 [0.90; 1.57]	508.53 (57.17)	478.32 (50.72)	30.21 [23.64; 36.78]	0.96 [0.70; 1.20]
Cities	496.42 (47.30)	466.51 (42.20)	29.90 [22.20; 37.61]	0.91 [0.63; 1.19]	514.59 (63.35)	499.62 (58.69)	14.97 [7.88; 22.06]	0.49 [0.24; 0.73]

SDs are presented in round brackets; Cohen’s d with 95% CI refers to the RT-CIT effect (difference between probes and irrelevants).

**Figure 1:**
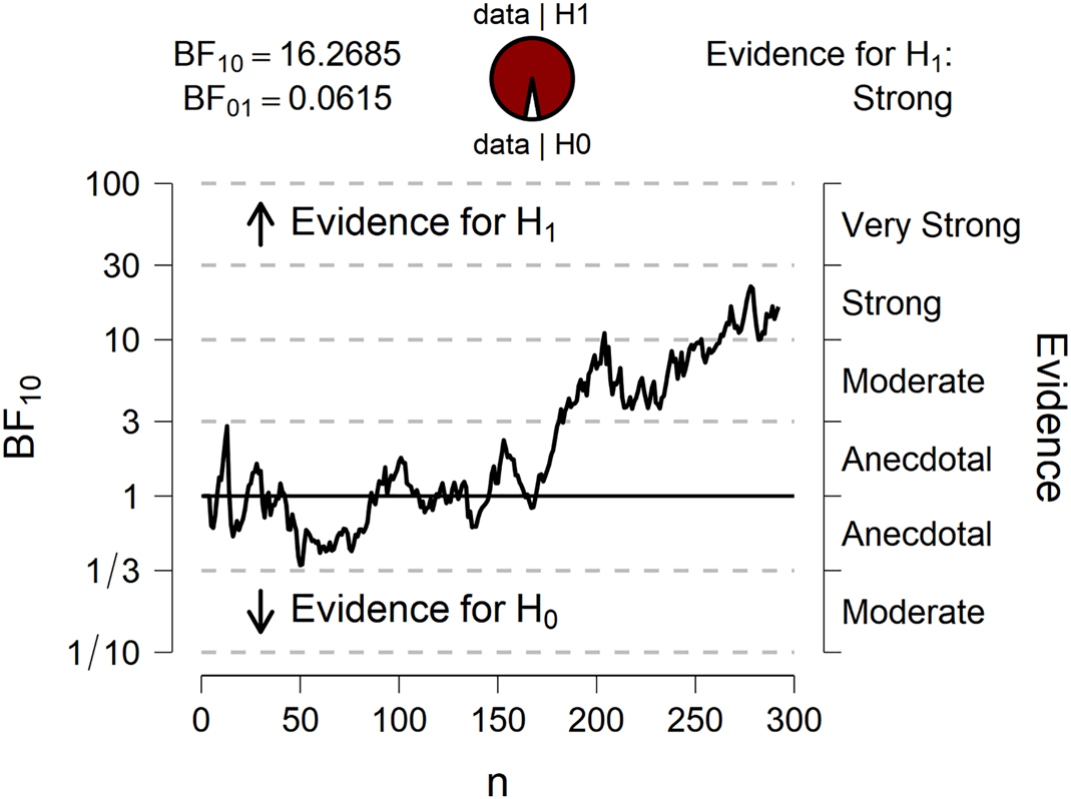
JASP output displaying a sequential analysis, showing the evidential flow of the alternative hypothesis (H_1_) versus the null hypothesis (H_0_), as data accumulates.

When analyzing only the first 2 batches of data, the data also supported our hypothesis, yet with a BF10 of 5.27. This is illustrated in Fig. 1, which shows the evidential flow as a function of increasing sample size (note that our data came in sequentially, in batches). Nevertheless, Bayesian one-sample *t* tests revealed that the CIT effects in each condition, separately for the two probe types, were significantly different from 0 (all BF10 > 215; all p’s < 0.001). Thus, while the RT-CIT effect was significantly attenuated in the no conflict condition compared to the conflict condition, it was significant in both conditions.

### Preregistered secondary analysis: ROCs

**Figure 2:**
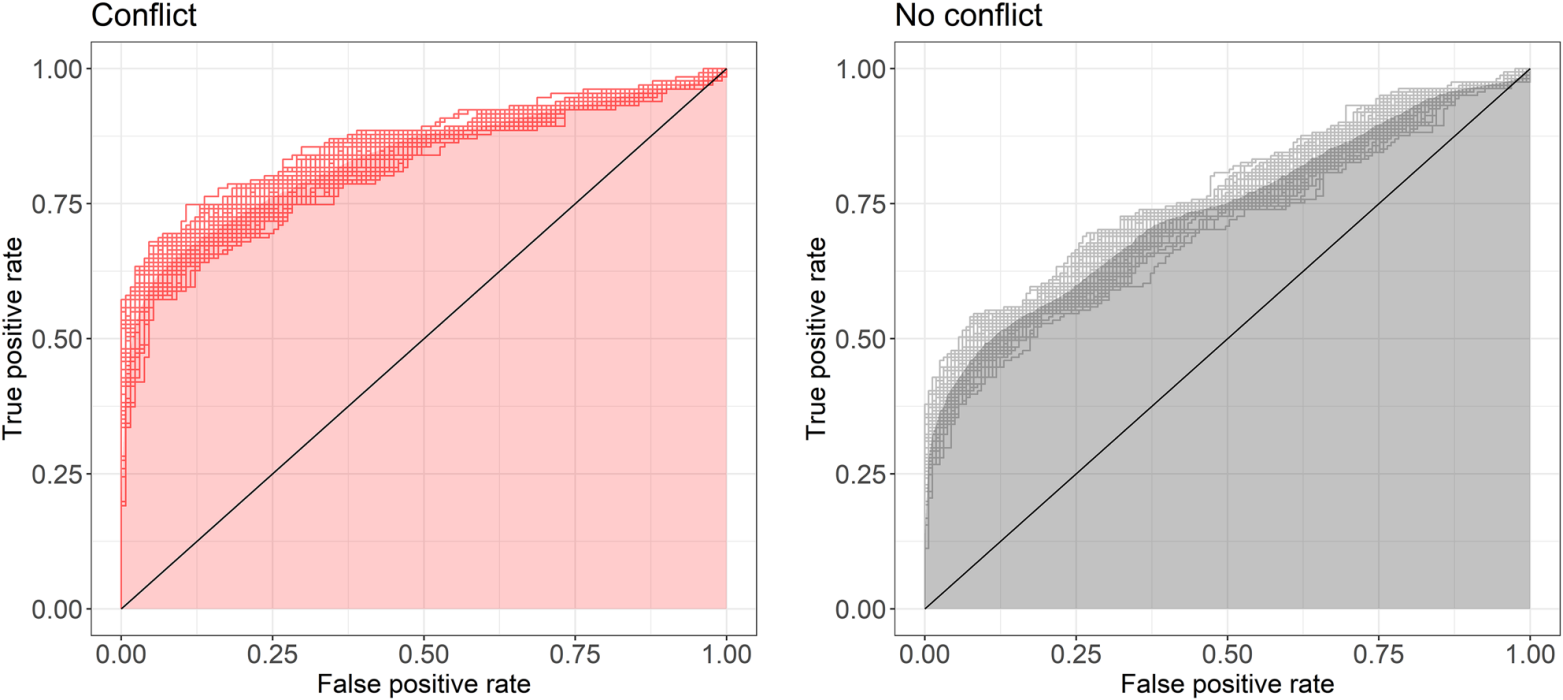
Mean AUC for the “conflict” and “no conflict” conditions. As the mean AUC values were based on 100 bootstrapped samples, this figure presents the mean AUC (i.e. the shaded area) as well as the 100 bootstrapped ROCs (i.e. the separate lines). The black diagonal line represents chance level.

In line with other RT-CIT studies and our pre-registration (https://aspredicted.org/e7ys3.pdf), we performed a ROC analysis. First, for each experimental condition, the area under the ROC curve (AUC) was calculated to measure the efficiency of classifying participants as naive or knowledgeable based on their individual probe-irrelevant score (i.e. the dCIT). The dCIT is the difference between the mean of probe RTs and the mean of irrelevant RTs, divided by the standard deviation of irrelevant RTs^32^. As there were no naïve (unknowledgeable) participants in this experiment, their data were simulated. This simulation procedure is based on the assumption that the probe items hold no special meaning for naïve participants and therefore there is no reason to expect that these items would elicit systematic differential RTs. Thus, for naïve participants, the expected mean value of dCIT is 0. The standard deviation of dCIT was estimated using the following formula: $\frac{N-1}{N-3}*\frac{4}{N}\left(1 \frac{{\delta }^{2}}{8}\right)$, where *N* is the total sample size and $\delta$ is the true effect size in the population, which is 0 in this case (e.g. Ref.^37^). Further, it was assumed that the data of individual naïve participants are distributed normally (see Ref.^38,39^). Hence, for each experimental condition, a simulated dataset was created by taking *n* random samples (where *n* equals either the number of participants in the conflict or no conflict condition) from the normal distribution (with a mean of 0 and a *SD* computed as explained above). This simulation procedure, as well as the computation of the AUC, were repeated 100 times using a bootstrapping procedure (both for the conflict and no conflict conditions). These 100 bootstrapped AUCs were then used to compute the mean AUC and its 95% confidence interval (CI). Accordingly, the mean AUC was 0.84 (95% CI [0.82, 0.86]) in the conflict condition and 0.74 (95% CI [0.71, 0.76]) in the no conflict condition. When statistically comparing these AUCs, using the formula suggested by Hanley and McNeil^40^, a significant difference was found: *Z* = 3.68, *p* < 0.001, BF_10_ = 53.78. In other words, the RT-CIT effect was significantly reduced in the *no conflict* condition as compared to the *conflict* condition (see Fig. 2). However, as the confidence interval of each condition does not include 0.5, the detection efficiency is significantly larger than chance even in the no conflict condition.

## Discussion

To strengthen the theoretical foundation of the CIT, the present study examined whether response conflict is sufficient to account for the RT-CIT effect. To this end, we compared a *conflict* condition with a *no conflict* condition. In the conflict condition, the response buttons emphasized familiarity (and participants pressed an “unfamiliar” button for familiar probes), while in the *no conflict* condition, the response buttons emphasized semantic categorical membership (and participants pressed either a “city” or “name” button for familiar probes). Our Bayesian analysis revealed that the RT-CIT effect (longer RTs to probes than irrelevants) was substantially stronger in the conflict condition than in the no conflict condition. Nevertheless, the RT-CIT effect was significantly larger than zero even in the no conflict condition.

These results are in line with previous RT-CIT studies, suggesting an important role for response conflict^16^. However, they also suggest that conflict is not the only contributing factor and that additional factors may contribute to the RT-CIT effect. One notable possibility is orientation to significant information. As explained previously, the orienting response is modulated by both stimulus significance and stimulus novelty^26,27^. Hence, as the probe items are significant (i.e. last name) and also novel (i.e. less frequently presented than the irrelevant items), even in the no conflict condition they should elicit an enhanced orienting response which may induce a brief interruption of ongoing activity and prolonged RTs^28,29^. The idea that probe items (i.e. concealed information items) capture attention and slow RT was tested by Verschuere et al.^41^. In this study, participants performed a “dot probe task” in which they had to try and conceal recognition of various studied pictures. Specifically, each of the studied pictures appeared on the screen together with an irrelevant (control) picture and after both pictures disappeared a pair of dots replaced one of them. Participants were instructed to indicate, as quickly as possible, whether the dots were positioned horizontally or vertically. As predicted, RTs were slower when the dots replaced probe (concealed) than irrelevant pictures. Yet, the observed effects were relatively small, presumably because the task did not induce any response conflict. Various other tasks, which should also be free of conflict, include the modified Stroop, the lexical decision, and the tone detection task. Importantly, all these tasks have produced similar results—i.e. relatively small RT differences between concealed and control information (see Verschuere & De Houwer^12^ for an in-depth discussion). Hence, together they support our findings which suggest that conflict is essential to produce a large and stable RT-CIT effect.

The present study joins previous theoretical work designed to shed light on the mechanisms that drive the CIT effect with different measures. These studies have relied on autonomic psychological measures, brain responses and ocular changes^15,23–25,36,42–44^. As the present study focuses on a behavioral measure (RT), it complements this earlier work and extends the theoretical framework of the CIT (see also Ref.^45^). Importantly, a better understanding of the theoretical basis of the CIT may also hold implications for the applied usage of the test. For example, as our results support the idea that conflict contributes to the RT-CIT effect, conflict should be maximized. This could be accomplished by increasing the reliance on familiarity-based responding^16^. For example, by including familiarity-related fillers, i.e. stimuli that are semantically related to the concept of “familiarity” and require the same binary classification as the other stimuli (e.g. the words ‘familiar’ and ‘unfamiliar’)^19^.

Future studies could further unravel the contribution of different mechanisms to the RT-CIT effect. For example, the possible role of orienting could be examined by comparing low salient and high salient items in a “no conflict condition”. If the RT-CIT is driven by conflict, as well as orientation, the RT-CIT effect should be minimized when conflict is removed and the probes are of low salience. However, how far can one minimize the RT-CIT effect? Note that even non-salient probes carry some level of salience (significance), otherwise one cannot define them as probes (low salient probes which have been used in previous studies include: one’s favorite ice cream, favorite animal, age, and academic major^24,32^). Hence, only future work can give a definite answer to these questions. Notably, in the current study we relied on two types of probe items: names (last name and mother’s name) and cities (favorite city and city of birth). Although both were expected to be highly salient, the two selected names were assumed to be most salient (which we cannot statistically prove as our significance ratings cannot be trusted, see below). Accordingly, the RT-CIT effect was substantially larger when names served as probes than when cities served as probes (BF_10_ = 22.52).

The present study has also limitations worth discussing. Mainly, our significance ratings were illogical. Participants rated the irrelevant items as more significant than the personal probes. Although this did not affect our RT results (in both conditions RTs were larger to probe items), it raises a question as to whether participants properly understood the instructions. As participants were not explicitly told to stop “concealing” after the CIT, it is possible that they were under the presumption that they still had to conceal by giving the probes lower ratings than the irrelevants. Since we cannot trust our significance ratings, we also cannot exclude the possibility that our conflict manipulation affected the significance of probes. As participants in the *conflict* condition had to deny familiarity with the probes, and consequently experienced a conflict, they might have found the probes more significant.

In sum, the present study was designed to determine whether response conflict is sufficient to account for the RT-CIT effect. Specifically, we compared a *conflict* with a *no conflict* condition and found the RT-CIT effect to be significantly reduced when conflict was removed. This suggests that response conflict drives at least part of the RT-CIT effect. However, as the RT-CIT effect remained significant and fairly large when conflict was removed, other mechanisms such as orienting may play a role as well. Taken together, these novel results will hopefully add to the expanding theoretical framework of the CIT and inspire new research designed to improve CIT validity.

## Data Availability

This study was preregistered (https://aspredicted.org/e7ys3.pdf). Materials, data, and analytic scripts have been made publicly available and can be accessed at https://osf.io/8p2b4/.
